# Biochar addition alleviate the negative effects of drought and salinity stress on soybean productivity and water use efficiency

**DOI:** 10.1186/s12870-020-02493-2

**Published:** 2020-06-22

**Authors:** Yaojun Zhang, Jiaqi Ding, Hong Wang, Lei Su, Cancan Zhao

**Affiliations:** 1 School of Life Sciences, Henan University, Kaifeng, 475004 Henan China; 2 College of Resource and Environment, Anhui Science and Technology University, No. 9 Donghua Road, Chuzhou, 233100 China

**Keywords:** Biochar, Crop productivity, Drought stress, Leaf gaseous exchange, Salinity stress, Water use efficiency

## Abstract

**Background:**

Environmental stress is a crucial factor restricting plant growth as well as crop productivity, thus influencing the agricultural sustainability. Biochar addition is proposed as an effective management to improve crop performance. However, there were few studies focused on the effect of biochar addition on crop growth and productivity under interactive effect of abiotic stress (e.g., drought and salinity). This study was conducted with a pot experiment to investigate the interaction effects of drought and salinity stress on soybean yield, leaf gaseous exchange and water use efficiency (WUE) under biochar addition.

**Results:**

Drought and salinity stress significantly depressed soybean phenology (e.g. flowering time) and all the leaf gas exchange parameters, but had inconsistent effects on soybean root growth and WUE at leaf and yield levels. Salinity stress significantly decreased photosynthetic rate, stomatal conductance, intercellular CO_2_ concentration and transpiration rate by 20.7, 26.3, 10.5 and 27.2%, respectively. Lower biomass production and grain yield were probably due to the restrained photosynthesis under drought and salinity stress. Biochar addition significantly enhanced soybean grain yield by 3.1–14.8%. Drought stress and biochar addition significantly increased WUE_-yield_ by 27.5 and 15.6%, respectively, while salinity stress significantly decreased WUE_-yield_ by 24.2%. Drought and salinity stress showed some negative interactions on soybean productivity and leaf gaseous exchange. But biochar addition alleviate the negative effects on soybean productivity and water use efficiency under drought and salinity stress.

**Conclusions:**

The results of the present study indicated that drought and salinity stress could significantly depress soybean growth and productivity. There exist interactive effects of drought and salinity stress on soybean productivity and water use efficiency, while we could employ biochar to alleviate the negative effects. We should consider the interactive effects of different abiotic restriction factors on crop growth thus for sustainable agriculture in the future.

## Background

Drought induced by water scarcity is a major limitation to the sustainability of global crop production [1, 2]. High frequency and severity of droughts have been predicted throughout the world in the future, including most parts of China, due to global warming and expected frequency of extreme climatic events [3–5]. Crop yield could be restrained by drought stress has been well documented in previous studies [6–10]. For instance, drought stress could significantly decrease soybean grain yield by 24–50% but gain higher water use efficiency (WUE) [11]. Meanwhile, WUE is an important trait for indicating plant resistance under drought stress [12].

Drought stress could alter physiological characteristics of plant leaves, such as lowering leaf photosynthetic and transpiration rate and stomatal conductance, thus restraining crop productivity [13–15]. In addition, drought stress could also affect plant phenology (e.g., advance or delay flowering time) and then influence crop productivity [16]. It has been reported that water shortage at flowering stage negatively affected chickpea (*Cicer arietinum* Linn.) yield [17], but soybean (*Glycine max* L.) flowering time was observed no response under drought stress [11]. Besides, root is always playing an important role in regulating crop productivity under drought stress, especially for the legume crop with nodules can fix N_2_ from atmosphere used as N nutrition [2, 18, 19]. For instance, plant always has deeper roots being able to assimilate more water and nutrition from deeper soil under drought stress [20].

Salinity is another one vital limiting factor for sustainable agriculture with depressing crop growth and production worldwide [21–23]. Globally, more than 70 countries have been characterized as existing large areas of salinity-affected lands and over 6% of the world’s total land is affected by salinity stress [24, 25]. Salinity stress could not only reduce crop yield through affecting leaf physiological growth [26], but also could reduce the ability of plant roots to take up water and nutrition (e.g., N) from soil [27, 28]. While other studies showed that salinity could increase transgenic barley growth and yield in both glasshouse and field conditions, but the mechanisms were unclear [29].

Biochar, a stable C-rich byproduct obtained from biomass, application to low fertility soils is a promising approach to improve soil quality and thus crop productivity [30–32]. Generally, biochar application could increase crop productivity mainly occurred in acidic and neutral pH soils [31, 33], but there were less studies on alkaline soil under drought stress.

Soybean, as one of the world’s most widely grown legume crops with a total production of more than 346 million tons in 2016 (FAO stat, http://www.fao.org/faostat/en/#data/QC), provides large amounts of protein and edible oil for human consumption [34]. This important legume crop, however, is affected by several abiotic stressors, such as drought and salinity, which could significantly restrict soybean growth and productivity [8, 11, 34, 35]. Previous studies have gained widely insight into the soybean productivity affected by drought and salinity, however, the physiological basis underlying the yield reduction is still not clear. In addition, whether biochar addition could be used as an effective management to infertile soil under the combination stress of drought and salinity is scarce. Thus, a better understanding of biochar addition on physiological basis and root traits for soybean growth under drought and salinity stress will be beneficial for sustainable agriculture.

Here, this study was carried out to examine biochar addition on soybean leaf physiological parameters, crop productivity and WUE at leaf and yield levels under the combination of drought and salinity stress. The aim of this study was to evaluate the single and interactive effect of these treatments on soybean productivity. This study have tested three main hypotheses: 1) drought stress could decrease soybean leaf physiological parameters and thus crop productivity, 2) salinity stress could aggravate the negative effects of drought stress, and 3) biochar addition could alleviate the constrain effects of drought and salinity stress.

## Results

### Soybean phenology and leaf gas exchange parameters

Soybean phenology (e.g. flowering time) and all leaf gas exchange parameters were significantly affected by drought and salinity stress, while no significant effect was observed as consequence of biochar addition, except for photosynthetic rate and stomatal conductance (Table 1). Drought stress at low and high intensity significantly decreased leaf photosynthetic rate by 26.3 and 37.9%, stomatal conductance by 38.9 and 55.0%, intercellular CO_2_ concentration by 15.8 and 17.1% and transpiration rate by 49.6 and 71.2%. On the contrary, drought stress significantly increased WUE_-leaf_ by 45.4 and 102.4% at D-L and D-H treatments, respectively. Soybean flowering time was delayed by almost 1 day under salinity stress. In addition, salinity stress significantly decreased photosynthetic rate, stomatal conductance, intercellular CO_2_ concentration and transpiration rate (− 20.7, − 26.3%, − 10.5% and − 27.2%, respectively) relative to the non-salinity treatment.

**Table 1:** Flowering time, *P*_-max_, Cond, Ci, Tr and WUE_-leaf_ of soybean at the flowering stage. Different letters within each treatment indicate significant differences for Fisher LSD test

	Flowering time	*P*_-max_	Cond	Ci	Tr	WUE_-leaf_
	days	μmol CO_2_ m^− 2^ s^− 1^	mol H_2_O m^− 2^ s^− 1^	μmol CO_2_ mol^− 1^	mmol H_2_O m^−2^ s^− 1^	μmol mmol^− 1^
Drought stress (D)						
D-C	41.75 ± 0.32 c	10.20 ± 0.68 a	0.49 ± 0.03 a	325.29 ± 7.27 a	11.69 ± 0.85 a	0.95 ± 0.09 c
D-L	42.92 ± 0.41 b	7.51 ± 0.47 b	0.30 ± 0.01 b	273.83 ± 4.90 b	5.89 ± 0.55 b	1.38 ± 0.08 b
D-H	44.54 ± 0.26 a	6.34 ± 0.21 b	0.22 ± 0.01 c	269.69 ± 6.16 b	3.37 ± 0.16 c	1.93 ± 0.06 a
Salinity stress (S)						
Control	42.58 ± 0.33 b	8.94 ± 0.56 a	0.38 ± 0.03 a	305.58 ± 7.17 a	8.08 ± 0.83 a	1.36 ± 0.09 a
Salinity	43.56 ± 0.32 a	7.09 ± 0.33 b	0.28 ± 0.02 b	273.62 ± 4.57 b	5.88 ± 0.63 b	1.48 ± 0.09 a
Biochar (B)						
B0	43.17 ± 0.40 a	7.02 ± 0.45 b	0.31 ± 0.03 b	289.18 ± 7.70 a	6.13 ± 0.76 a	1.38 ± 0.11 a
B1	42.96 ± 0.41 a	8.47 ± 0.75 a	0.35 ± 0.04 a	285.04 ± 7.67 a	7.42 ± 1.00 a	1.39 ± 0.11 a
B2	43.08 ± 0.42 a	8.55 ± 0.48 a	0.34 ± 0.03 ab	294.58 ± 7.85 a	7.40 ± 1.01 a	1.50 ± 0.12 a
ANOVA						
D	**< 0.001**	**< 0.001**	**< 0.001**	**< 0.001**	**< 0.001**	**< 0.001**
S	**< 0.01**	**< 0.001**	**< 0.001**	**< 0.001**	**< 0.01**	0.055
B	0.896	**< 0.01**	**< 0.05**	0.369	0.156	0.274
D × S	0.057	**< 0.01**	**< 0.001**	0.079	0.141	**< 0.001**
D × B	0.550	0.562	0.174	0.146	0.164	0.070
S × B	0.469	**< 0.05**	0.094	**< 0.05**	0.401	**< 0.001**
D × S × B	0.328	**< 0.05**	**< 0.05**	0.156	0.717	0.339

Note: *P*_*-max*_, leaf maximum photosynthetic rate; Cond, stomatal conductance; Ci, intercellular CO_2_ concentration; Tr, transpiration rate; WUE_-leaf_, leaf water use efficiency

**Fig. 1:**
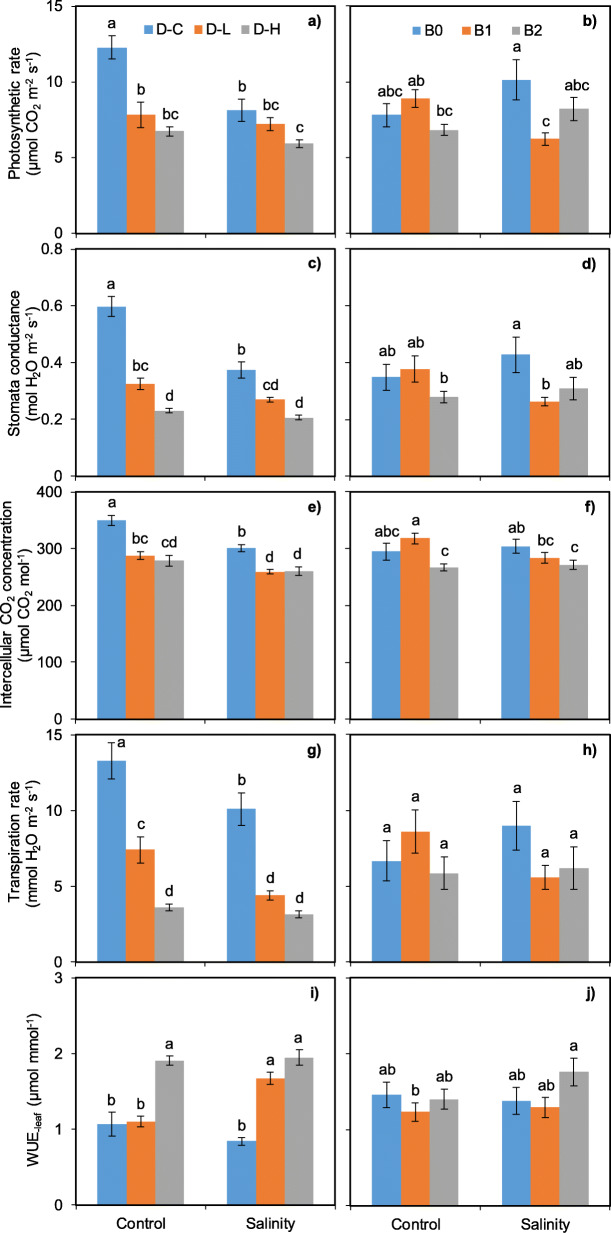
Leaf photosynthetic rate (**a**, **b**), stomatal conductance (**c**, **d**), intercellular CO_2_ concentration (**e**, **f**), transpiration rate (**g**, **h**) and WUE_-leaf_ (**i**, **j**) as affected by the interactive effects of drought × salinity stress and biochar × salinity stress. D-C = no drought stress; D-L = low drought stress; D-H = high drought stress. The bars indicate the standard error of the means (±SE). Different letters indicate significant differences according to the Fisher’s LSD test

The present study showed few interactive effects of treatments on leaf gas exchange parameters and no effect on soybean flowering time (Table 1, Fig. 1). Photosynthetic rate and stomatal conductance were significantly influenced by interactions both drought × salinity and drought × salinity × biochar. Intercellular CO_2_ concentration was significantly affected only by salinity × biochar addition interaction. WUE_-leaf_ showed significant changes considering drought × salinity and salinity × biochar addition interaction.

### Biomass production

Drought and salinity stress significantly affected soybean biomass productivity and root growth (Table 2). With drought stress increasing, shoot biomass (− 28.9% and − 48.3% at D-L and D-H, respectively), root biomass (− 4.7% and − 34.3% at D-L and D-H, respectively) and total biomass (− 25.5% and − 46.3% at D-L and D-H, respectively) were depressed significantly compared with the control. On the contrary, drought stress significantly increased root length (21.7 and 10.6% at D-L and D-H, respectively) compared with the control and the longest root length occurred in D-L treatment.

**Table 2:** Shoot biomass, root biomass, shoot/root, total biomass, root length, grain yield and WUE_-yield_ of the soybean plant when harvesting. Different letters within each treatment indicate significant differences for Fisher LSD test

	Shoot biomass	Root biomass	Shoot/root	Total biomass	Root length	Grain yield	WUE_-yield_
	g pot^−1^	g pot^−1^		g pot^−1^	cm	g pot^−1^	g L^−1^
Drought stress (D)							
D-C	19.80 ± 1.21 a	3.24 ± 0.25 a	6.35 ± 0.30 a	23.04 ± 1.40 a	28.08 ± 1.72 b	10.46 ± 0.64 a	0.51 ± 0.03 b
D-L	14.08 ± 0.85 b	3.08 ± 0.18 a	4.66 ± 0.21 b	17.17 ± 0.98 b	34.17 ± 1.74 a	8.61 ± 0.61 b	0.65 ± 0.05 a
D-H	10.23 ± 0.74 c	2.13 ± 0.15 b	4.96 ± 0.29 b	12.36 ± 0.86 c	31.04 ± 2.37 ab	6.00 ± 0.29 c	0.64 ± 0.03 a
Salinity stress (S)							
Control	15.56 ± 0.74 a	3.21 ± 0.12 a	4.90 ± 0.18 b	18.76 ± 0.82 a	34.88 ± 1.83 a	9.34 ± 0.46 a	0.69 ± 0.03 a
Salinity	13.86 ± 1.22 a	2.42 ± 0.21 b	5.75 ± 0.29 a	16.28 ± 1.39 b	27.31 ± 1.12 b	7.37 ± 0.55 b	0.52 ± 0.03 b
Biochar (B)							
B0	12.33 ± 0.82 b	2.43 ± 0.17 b	5.20 ± 0.27 a	14.76 ± 0.96 b	31.80 ± 2.01 a	7.89 ± 0.48 b	0.57 ± 0.04 b
B1	14.09 ± 1.03 b	2.82 ± 0.19 ab	5.09 ± 0.29 a	16.91 ± 1.16 b	33.17 ± 2.29 a	8.13 ± 0.65 ab	0.59 ± 0.04 b
B2	17.70 ± 1.53 a	3.20 ± 0.28 a	5.68 ± 0.35 a	20.90 ± 1.74 a	28.31 ± 1.60 a	9.05 ± 0.78 a	0.66 ± 0.03 a
ANOVA							
D	**< 0.001**	**< 0.001**	**< 0.01**	**< 0.001**	**< 0.05**	**< 0.001**	**< 0.001**
S	0.051	**< 0.001**	**< 0.01**	**< 0.05**	**< 0.001**	**< 0.001**	**< 0.001**
B	**< 0.001**	**< 0.01**	0.438	**< 0.001**	0.090	**< 0.05**	**< 0.05**
D × S	0.059	**< 0.05**	0.820	**< 0.05**	**< 0.05**	**< 0.001**	**< 0.001**
D × B	**< 0.05**	0.401	0.534	**< 0.05**	**< 0.05**	**< 0.001**	**< 0.001**
S × B	**< 0.05**	**< 0.05**	0.853	**< 0.05**	0.714	**< 0.01**	**< 0.01**
D × S × B	0.682	0.499	0.716	0.633	**< 0.05**	0.160	0.160

Salinity stress significantly decreased root biomass (− 24.5%) and total biomass (− 13.2%) relative to control treatment. In accordance with root biomass, salinity stress significantly decreased root length by 21.7% compared with control.

Biochar addition showed significantly effects on shoot biomass, root biomass and total biomass, but had no effect on the ratio of shoot/root and root length (Table 2). With biochar addition rate increasing, higher shoot biomass (14.3 and 43.6% at B1 and B2, respectively), root biomass (15.8 and 31.5% at B1 and B2, respectively) and total biomass (14.6 and 41.6% at B1 and B2, respectively) than control were observed.

**Fig. 2:**
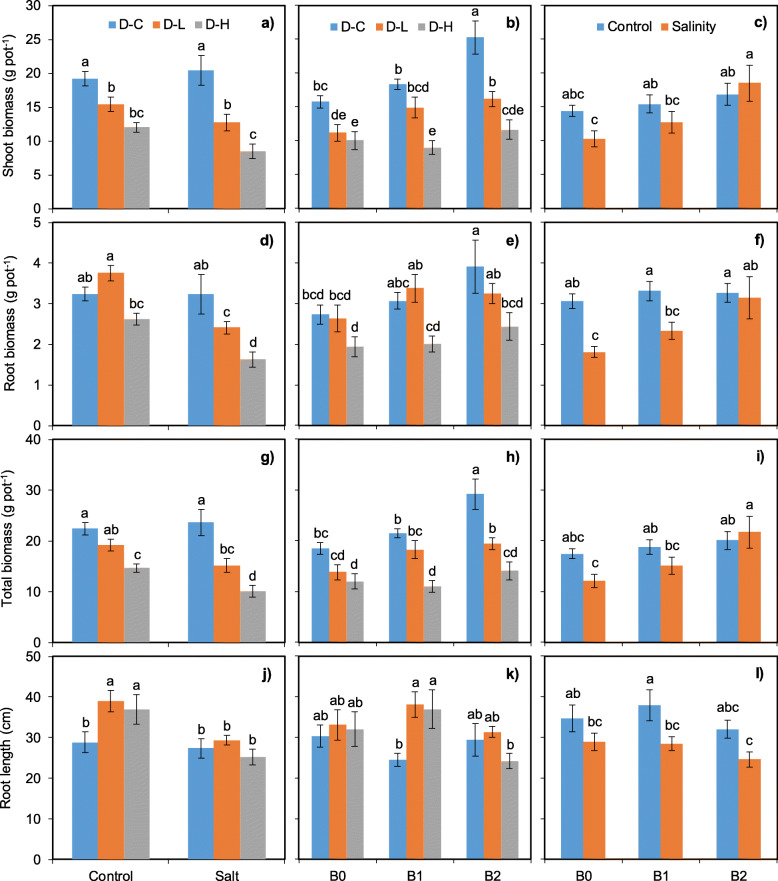
Shoot biomass (**a**, **b**, **c**), root biomass (**d**, **e**, **f**), total biomass (**g**, **h**, **i**) and root length (j, k, l) as affected by the interactive effects of drought and salinity stress as well as biochar addition. D-C = no drought stress; D-L = low drought stress; D-H = high drought stress. The bars indicate the standard error of the means (±SE). Different letters indicate significant differences according to the Fisher’s LSD test

Generally, biomass production was partially affected by the interactive effects of drought stress, salinity stress and biochar addition (Table 2). Specifically, drought × salinity stress interaction significantly affected root length, root biomass and the total biomass production (Fig. 2). It is worth mentioning that root length showed no difference among drought stress when salinity was added, but without salinity addition root length was enhanced by 35.5% under D-L and 28.1% under D-H compared to D-C treatment (Fig. 2j). In addition, the drought stress × biochar addition interaction significantly affected shoot biomass, total biomass, and root length but not root biomass. With biochar addition increasing, drought stress depressed shoot biomass (averaged from − 19.0% to − 53.8%) and total biomass (averaged from − 14.8% to − 51.7%) stronger compared with control. Particularly, drought stress significantly increased root length (55.2 and 50.6% at low and high drought stress, respectively) only in B1 treatment.

### Grain yield

Soybean gained the highest grain yield (10.46 g pot^− 1^) at the D-C treatment with well irrigation. Drought stress significantly reduced the grain yield of soybean by 17.7 and 42.6% under low and high drought, respectively (Table 2). Similarly, salinity stress significantly lowered the grain yield by 21.1% compared with the treatment with no salinity addition. While, biochar addition significantly enhanced grain yield by 3.1–14.8% compared with the control.

**Fig. 3:**
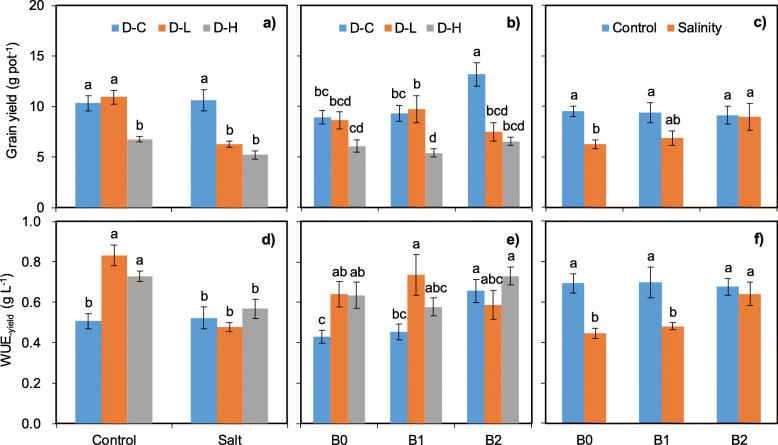
Soybean grain yield (**a**, **b**, **c**) and WUE_-leaf_ (**d**, **e**, **f**) as affected by the interactive effects of drought and salinity stress as well as biochar addition. D-C = no drought stress; D-L = low drought stress; D-H = high drought stress. The bars indicate the standard error of the means (±SE). Different letters indicate significant differences according to the Fisher’s LSD test

Soybean grain yield was partially affected by the interactive effects of studied treatments (Table 2). As expected, drought × salinity stress interaction significantly affected grain yield with worse performance when salinity was added together with drought stress (Fig. 3). Besides, drought stress interaction with biochar addition also significantly affected soybean grain yield. No significant effect on soybean yield was observed considering the interaction of drought stress × salinity stress × biochar addition.

### WUE_-yield_

Drought stress showed a positive effects on WUE_-yield_ while salinity stress showed a negative effects on WUE_-yield_ in this study (Table 2). Under water deficit, WUE_-yield_ was increased by 27.5 and 25.5% under low and high drought stress, respectively. On the contrary, salinity stress significantly decreased WUE_-yield_ by 24.2% compared with the non-salinity addition soils. Biochar addition significantly enhanced WUE_-yield_ 15.6% at high addition rate while showed no effect at low addition rate.

WUE_-yield_ was significantly affected by the studied treatments interaction (Table 2, Fig. 3). Drought stress significantly increased WUE_-yield_ but salinity addition significantly decreased WUE_-yield_ under control treatment, however, biochar addition relieved the effects.

## Discussion

Flowering stage is an important transition period for soybean vegetative and reproductive growth, and sensitive to drought and salinity stress [36]. Both drought and salinity stress delayed soybean flowering time in this study, which should be an underlying mechanism for soybean adapting to the rigorous habitat. Previous studies have shown that drought and salinity stress could delay crop flowering time thereby making a negative effect on crop productivity [11, 21, 37, 38]. The present study is in consistent with the above reported literature findings, which might be attributed to the greater water consumption with later flowering time [11].

Leaf photosynthetic efficiency plays an important role in regulating crop yield [15, 35, 39]. The present study showed that leaf photosynthetic rate was inhibited by drought and salinity stress, which could cause reduction of soybean yield [15, 16]. The decrease of photosynthetic rate due to drought stress has been also reported in legume crops [15, 40], and has been ascribed to stomatal closure under drought stress [14, 15, 59]. As reported by Hussain et al. [15], stomatal closure mediated restricted CO_2_ diffusion in the leaves is more dominating compared to CO_2_ assimilation, thus could decline leaf photosynthetic rate and crop productivity. In accordance with previous studies, the reduction of photosynthetic rate in the present study can be ascribed to two distinct mechanisms: 1) through decreased CO_2_ diffusion within the leaf due to stomatal closure (decreased by 56.7 and 80.3% under low and high drought stress, respectively). 2) through decreased the enzyme at the acceptor site of ribulose-1, 5-bisphosphate carboxylase/oxygenase or inhibit photosynthetic enzymes due to lower intercellular CO_2_ concentration [16, 40, 41]. Similarly, the intercellular CO_2_ concentration is also considered as a key factor assessing the effects of salinity on photosynthetic efficiency [21, 42]. Soil salinity stress could lead to enhance leaf cellular Na^+^ and Cl^−^ concentrations, then depress cell expansion and photosynthetic activity and thereafter accelerate leaf senescence, thus resulting in crop yield reduction [21, 43]. Otherwise, salinity tolerant plant showed a better intercellular CO_2_ concentration in the leaf for the photosynthetic rate [23].

Root, the first organ to adapt and respond sensitively to abiotic stressors in soil (e.g., drought and salinity), plays an important role in regulating plant growth [44–46]. To our knowledge, however, few work has been done on root and nodule growth of soybean responding to the interaction of drought and salinity stress. Root architecture, particularly those that can entrench deeper with longer root length in the soil, plays an important role in maximizing the ability of plants to gain soil water and nutrients for plant growth [44, 47]. Accompanied with strategies that reduce water loss, such as stomatal closure and leaf transpiration rate weaken, the augment in root length could increase soil water and nutrients obtain that is necessary to support biomass production and grain yield of soybean [44]. As shown in this study, root length was longer under drought condition than the control for acquiring more water and nutrition easier. On the contrary, root nodules were decreased sharply accompanied with soybean grain yield under drought and salinity stress. These findings suggest that in addition to root performance, the ability to develop and maintain root nodules may also be a crucial trait regulating the grain productivity of soybean. Although salinity addition showed no effect on root length and nodule weight, the interaction of drought and salinity suggests that we should consider the comprehensive influence of salinity on soybean productivity under drought stress, because root growth and nodule performance are crucial parameters associated with soybean productivity.

Biochar application to low fertility or pollutant soils as a promising approach to improve soil quality and thus enhance crop yield has been well reported in previous studies [30–32, 48]. Actually, biochar application in field could enhance crop yield mainly ascribed to the regulation in soil pH [49], increase soil C storage [50], and retain soil water and nutrient [51]. In the present study, biochar addition significantly enhanced shoot biomass, root biomass and grain yield. These phenomenon could also be partially ascribed to the alternation in leaf physiological variables (e.g. photosynthesis). For example, biochar addition significantly enhanced soybean leaf photosynthetic rate and stomatal conductance (Table 1), this could give rise to a positive effect on leaf and soil available N content and thus increase soybean grain yield [52].

In rain-fed and semiarid regions, WUE has been regarded as an important trait indicating crop productivity, which links water and nutrient cycling in agroecosystems [39]. However, few studies have focused on how the WUE responded to drought and salinity stress at different scales, such as at leaf and yield levels. Drought and salinity stress showed the same effect on enhancing WUE at leaf scale in the present study (Table 1). The positive effect of drought and salinity on WUE_-leaf_ is largely due to leaf stomatal closure and transpiration rate reduction under the external stress [53, 54], thus leads to water evaporation less and water use more efficiency for the leaf. However, at the yield scale, WUE_-yield_ was enhanced significantly by drought stress but decreased significantly by salinity stress. The inversed results were probability caused by root growth responded differently to drought and salinity stress, which is sensitively to obtain water from soil [55]. Furthermore, the interactive of drought and salinity stress significantly affected WUE at both leaf and yield levels, which means we should consider the comprehensive influence of drought and salinity on WUE in the future for sustainable agriculture.

## Conclusions

This study shows that both drought and salinity stress delayed soybean flowering time and depressed leaf gas exchange parameters (e.g., photosynthetic rate, stomatal conductance, intercellular CO_2_ concentration and transpiration rate) with negative effect on grain yield. Biochar addition significantly increased plant biomass and grain yield. Drought stress showed an increase of WUE_-leaf_ and WUE_-yield_ while salinity stress showed a reduction of WUE_-yield_. Effective use of water implies maximal soil moisture capture for transpiration, which may be use to replace WUE in the future with drought stress. The results of this study indicate that drought and salinity stress effect on soybean productivity and WUE are highly conspicuous, while biochar amendment could alleviate the negative effects. We should take into account the employment of biochar and interactive effects of abiotic stressors for sustainable agriculture in the future.

## Methods

### Soil sampling and biochar description

Soil samples (0–20 cm depth cores) were collected from a sandy-loam vertisol (USDA soil classification system) managed with maize (*Zea mays* L.) and wheat (*Triticum aestivum* L.) crop rotation at the Research and Education Farm of Henan University, China (34° 49′ N, 114° 17′ E, 73 m a.s.l). The mean annual temperature is 14.5 °C, with monthly mean temperature ranging from − 0.16 °C in January to 27.1 °C in July (China Meteorological Data Sharing Service System). Mean annual precipitation is 627 mm, with 87.8% distributing from April to October. The soil parent material is mainly formed from Yellow River sediment, consisting of 65.6% sand, 14.1% silt and 20.3% clay with an initial pH of 8.6 (1:2.5, water/soil, w/w) and an average bulk density of 1.35 g cm^− 3^. Total N and organic C contents were 0.47 g kg^− 1^ and 11.04 g kg^− 1^, respectively. The electrical conductivity of saturated soil-paste extract (ECe) is 10.6 dS m^− 1^.

Biochar used in this study was produced from wheat straw under pyrolysis temperature of 550 °C at the Sanli New Energy Company in Henan, China. The main properties of biochar were reported in our previous study [56]: total organic C 467.2 g kg^− 1^, total N 6 g kg^− 1^, pH 10.9 and ash content 20.8%.

### Experimental design

A 3 × 2 × 3 factorial design pot experiment was conducted with the following main factors: 1) drought stress (main factor): soil moisture was kept at 75–80% WHC as control (D-C), 40–45% WHC as low drought stress (D-L), 20–25% WHC as high drought stress (D-H); 2) salinity stress (secondary factor): background soil as control and salinity addition at 1 g kg^− 1^ dry soil as the salinity stress treatment; 3) biochar addition (thirdly factor): biochar applied at 0, 5, and 10 g kg^− 1^ soil as control (B0), low (B1) and high (B2) biochar addition rate, respectively. In total, there were eighteen treatment combinations replicated four times for a total of 72 pots. In each plastic pot (with a circle radius of 20 cm and height of 25 cm), 5.6 kg soil (air dried weight basis) was added and soil surface was subsequently levelled before soybean sowing. The pot experiment was carried out in a rain shelter covered with glass.

Drought stress was controlled based on soil moisture with an electronic balance after thinning seedlings. Every 1 or 2 days, experiment pots were weighted and distilled water was used to replenish water loss if it was necessary. Salinity stress was adjusted by mixing NaCl into soil. Na^+^ content was 0.03 g kg^− 1^ in the background soil, but we refer to the background soil as the control treatment as none Na^+^. NaCl and biochar was mixed thoroughly with soil prior to experiment start according to pre-determined amount.

The pot experiment was lasted 107 days, it began on June 30 and ended on October 15 in 2018. The soybean variety (Named Zhonghuang 35, produced by Anhui Mindeli Seed Co. LTD) used in this experiment was one of the most widely planted variety in this region. Six well-selected soybean seeds were sowing in each pot. Thinning seedlings occurred after 20 days of sowing when the soybean plants have 3 or 4 cotyledons and two soybean plants were remained in each pot. Drought stress started after thinning seedlings.

### Leaf gas exchange measurements

The third leaf from the top plant was used to determine the leaf photosynthetic physiology at soybean flowering stage, including photosynthetic rate (*P*_*-max*_), stomatal conductance (Cond), intercellular CO_2_ concentration (Ci) and transpiration rate (Tr). The leaf photosynthetic physiology parameters was measured (two times on August 4, 2018 and August 11, 2018) by using an open gas-exchange system (Li-6400; Li-Cor Inc.) in the cloudless day between 08:00 to 11:00 (local time). During the measurement, leaves were illuminated at 1500 μmol m^− 2^ s^− 1^ using the LED light system. We did not intervene environmental changes, including leaf temperature, water vapor or CO_2_ concentrations. Leaf level WUE (WUE_-leaf_) was calculated as: WUE_-leaf_ = *P*_*-max*_ / Tr [57].

### Grain yield, biomass and WUE at yield level (WUE_-yield_) measurement

Grain yield from each pot was collected in mesh bags and air-dried for weighing. At the same time, soybean shoot samples were taken from each pot and oven-dried at 70 °C to constant weight for calculating the shoot biomass. For the soybean root, we have washed the root cleanly and measured root length.

WUE at the yield level was calculated by dividing the soybean grain yield by water usage [58]:

$$ {\mathrm{WUE}}_{-\mathrm{yield}}\left(\mathrm{g}\ {\mathrm{L}}^{-1}\right)=\mathrm{grain}\ \mathrm{yield}/\mathrm{water}\ \mathrm{usage} $$

### Data analysis and statistics

Leaf gas exchange parameters, soybean grain yield, biomass, root length and WUE were analyzed with three-way ANOVA and significant differences were checked through Fisher LSD test. All the parameters as affected by the interactive of drought stress, salinity stress and biochar addition were addressed in the figures. Statistical analysis of data was performed using SPSS version 21.0 (SPSS Inc.), and statistical significance was determined at the 0.05 probability level. The data are presented as means ± SE (*n* = 4).

## Data Availability

The datasets used and/or analysed during the current study are available from the corresponding author on reasonable request.
